# Cross-effect of TRPV1 and EP3 receptor on coughs and bronchopulmonary C-neural activities

**DOI:** 10.1371/journal.pone.0246375

**Published:** 2021-02-02

**Authors:** Xiuping Gao, Jianguo Zhuang, Lei Zhao, Wan Wei, Fadi Xu

**Affiliations:** 1 Pathophysiology Program, Lovelace Biomedical Research Institute, Albuquerque, New Mexico, United States of America; 2 Department of Exercise Physiology, Beijing Sport University, Beijing, China; 3 Dongfang Hospital Affiliated to Beijing University of Chinese Medicine, Beijing, China; Indiana University School of Medicine, UNITED STATES

## Abstract

Prostaglandin E_2_ (PGE_2_)-induced coughs *in vivo* and vagal nerve depolarization *in vitro* are inhibited by systemic and local administration of prostaglandin EP3 receptor (L-798106) and TRPV1 antagonists (JNJ 17203212). These results indicate a modulating effect of TRPV1 on the EP3 receptor-mediated cough responses to PGE_2_ likely through the vagal sensory nerve. This study aimed to determine whether 1) inhalation of aerosolized JNJ 17203212 and L-798106 affected cough responses to citric acid (CA, mainly stimulating TRPV1) and PGE_2_; 2) TRPV1 and EP3 receptor morphologically are co-expressed and electrophysiologically functioned in the individual of vagal pulmonary C-neurons (cell bodies of bronchopulmonary C-fibers in the nodose/jugular ganglia); and 3) there was a cross-effect of TRPV1 and EP3 receptor on these neural excitations. To this end, aerosolized CA or PGE_2_ was inhaled by unanesthetized guinea pigs pretreated without or with each antagonist given in aerosol form. Immunofluorescence was applied to identify the co-expression of TRPV1 and EP3 receptor in vagal pulmonary C-neurons (retrogradely traced by DiI). Whole-cell voltage patch clamp approach was used to detect capsaicin (CAP)- and PGE_2_-induced currents in individual vagal pulmonary C-neurons and determine the effects of the TRPV1 and EP3 receptor antagonists on the evoked currents. We found that PGE_2_-induced cough was attenuated by JNJ 17203212 or L-798106 and CA-evoked cough greatly suppressed only by JNJ 17203212. Approximately 1/4 of vagal pulmonary C-neurons co-expressed EP3 with a cell size < 20 μm. Both CAP- and PGE_2_-induced currents could be recorded in the individuals of some vagal pulmonary C-neurons. The former was largely inhibited only by JNJ 17203212, while the latter was suppressed by JNJ 17203212 or L-798106. The similarity of the cross-effect of both antagonists on cough and vagal pulmonary C-neural activity suggests that a subgroup of vagal pulmonary C-neurons co-expressing TRPV1 and EP3 receptor is, at least in part, responsible for the cough response to PGE_2_.

## Introduction

Cough is an important respiratory defense mechanism and one of the most common symptoms in clinical settings [1–5]. It is triggered primarily by stimulating vagal unmyelinated C-fibers and myelinated Aδ fibers innervating the airways [6, 7]. Cell bodies of these fibers reside in the nodose and jugular ganglia [8] and make synapses mainly in the nucleus tractus solitarius (NTS), where the second-order neurons further project to the respiratory center to reflexively elicit cough [9, 10]. Bronchopulmonary C-fibers (PCFs) in the vagal afferents are involved in the cough responses to aerosolized capsaicin (CAP) or citric acid (CA), primarily through acting on transient receptor potential cation channel subfamily V member 1 (TRPV1) [10–13]. On the other hand, prostaglandin E_2_ (PGE_2_), a common inflammatory mediator, stimulates vagal sensory fibers to evoke cough via acting on prostaglandin EP3 receptor [14–17]. TRPV1 is a Ca^2+^ permeable non-selective cation channel and activated by exogenous and endogenous ligands including CAP, CA, low pH, resinoferotoxin, lipoxygenase products, anandamide and HPETE [18–22]. The EP3 receptor as a PGE_2_ receptor, same as the other types of EP receptor (EP1-2 and EP4), is a G-protein-coupled receptor (GPCR) [23] and specifically contributes to the cough through activating airway sensory nerves [24, 25]. For example, PGE_2_ evokes responses in primary neurons isolated from sensory ganglia (using calcium imaging) and vagal sensory nerves via EP3 receptor in guinea pigs [26]. Clinically, elevation of both pulmonary endogenous TRPV1 stimulants and PGE_2_ has been believed to be responsible for coughs in patients with asthma, idiopathic pulmonary fibrosis, and COPD [27]. Moreover, PGE_2_-induced cough is also affected by blockade of TRPV1 (for review, see [28]). These reports point to a cross-effect of TRPV1 and EP3 receptor on cough response. In fact, PGE_2_ has been reported to be able to sensitize the dissociated vagal sensory neural response to CAP via EP2 receptor in rats [29, 30]. However, it is not yet clear whether TRPV1- and EP3 receptor-mediated response occurs in the same individual of pulmonary C-neurons contributing to the cough response and, if so, whether there is a cross-effect between TRPV1 and EP3 receptor on the neural excitation.

The evidence is accumulating to support the cross-effect of TRPV1 and EP3 receptor on the cough generation and vagal sensory nerve excitation. First, intraperitoneal (IP) injection of the TRPV1 or EP3 receptor antagonist strikingly reduces PGE_2_-evoked cough in guinea pigs. In parallel, local administration of the TRPV1 antagonist decreases PGE_2_-induced vagal nerve depolarization in humans, guinea pigs and mice *in vitro* [14, 26]. Second, pre-inhalation of PGE_2_ enhances the sensitivity of cough response to inhalation of CAP in healthy humans [27]. Third, PGE_2_ via activation of EP3 receptors can induce intracellular Ca^2+^ increase [31, 32], similar to intracellular Ca^2+^ elevation by TRPV1 activation [33]. Fourth, morphological studies have shown that vagal pulmonary C-neurons abundantly express TRPV1 [34, 35] and that nodose ganglion neurons express EP3 receptor [36, 37]. Collectively, to date, it is still unknown whether TRPV1 and EP3 receptor are co-expressed in vagal pulmonary C-fibers (C-neurons) and whether there is a cross-effect of TRPV1 and EP3 receptor occurring in these neurons and therefore contributing to the cough response.

To answer these questions, four series studies were carried out in this study to test whether: (1) inhalation of TRPV1 or EP3 receptor antagonist, similar to IP injection previously reported [14, 26], affects CA- and PGE_2_-induced coughs in unanesthetized guinea pigs; (2) TRPV1 and EP3 receptor are co-expressed in vagal pulmonary C-neurons; (3) CAP- and PGE_2_-induced currents in vagal pulmonary C-neurons are modulated by TRPV1 and EP3 receptor; and (4) PGE_2_ facilitates CAP-induced currents via activation of EP3 receptor and the intracellular signaling pathway of protein kinase A (PKA), while CAP affects PGE_2_-induced currents via TRPV1 *in vitro*. Our results suggest that a subgroup of vagal pulmonary C-neurons co-expressing TRPV1 and EP3 receptor is, at least in part, responsible for the cross-effect of TRPV1 and EP3 receptor on the cough response to PGE_2_.

## Materials and methods

### Ethics statement

All animals were managed in accordance with the Guide for the Care and Use of Laboratory Animals and approved by the Institutional Animal Care and Use Committee (IACUC), which is accredited by the Association for Assessment and Accreditation of Laboratory Animal Care International, USA (protocol# FY18-060).

### Animals

A total of 92 pathogen-free male Dunkin-Hartley guinea pigs (200–250 g, Charles River Laboratories, Inc. Wilmington, MA) were ordered and quarantined for 7 days before the experiments. Animals had access to food and water ad libitum with temperature and humidity ranged from 22–26°C and 30–65%. After quarantine, the animals were individually placed in a whole-body, unrestrained, plethysmograph chamber (model PLY3215, Buxco Electronics Inc., Troy, NY) for habituation (10, 20, and 40 min for day 1, 2, and 3 respectively before the cough test). After habituation, the animals were used for Study Series I as described below.

#### Intratracheal instillation of DiI

The animal was anesthetized by isoflurane (3–5%) to sufficiently suppress corneal and withdrawal reflexes and received intratracheal instillation (0.05 ml X 2) of DiI (1,1’-dioctadecyl-3,3,3’,3’-tetramethylindocarbocyanine perchlorate, 0.25 mg/ml, 1% ethanol concentration) to retrogradely trace vagal pulmonary C-neurons within the nodose and jugular ganglia. As previously reported [38], ten to twelve days later, the animals were euthanized to collect the ganglia for either immunocytochemical or biologically identifying vagal pulmonary sensory neurons.

### Experimental protocols

#### Series I—To test the effects of inhaling TRPV1 or EP3 receptor antagonist on CA- or PGE_2_-induced coughs

After adaptation, the animals were individually placed in the chamber again. Following stabilization for at least 5 min, they were exposed to vehicle (saline containing 1% DMSO for both JNJ 17203212 and L-798106), TRPV1 antagonist (JNJ 17203212, 1.5 mg/ml) and EP3 receptor antagonist (L-798106, 1.5 mg/ml) for 5 min respectively in three groups (n = 14/group). Then a 10 min exposure to aerosolized CA (150 mM) was performed in half of the animals in each group (n = 7) and to PGE_2_ (0.43 mM) in the remaining animals. The doses of CA and PGE_2_ were the same as that previously reported [39] and those of inhaling JNJ 17203212 and L-798106 were calculated based on the concentration of IP injection as well as the EC_50_ value of JNJ 17203212 and L-798106 *in vitro* [14, 26, 40]. CA rather than CAP was chosen here for the cough study *in vivo* because CAP, different from CA [39], failed to constantly evoke a cough response with greater individual variation in guinea pigs [26].

#### Series II—To immunohistochemically identify co-expression of TRPV1 and EP3 receptor in vagal pulmonary neurons

Four guinea pigs were utilized initially to immunohistochemically assess the co-expression of TRPV1 and EP3 receptor in nodose and jugular ganglion neurons. After euthanasia and fixation, the nodose and jugular ganglia were harvested and prepared for immunocytochemically labeling TRPV1 and EP3 receptor. Because EP3 receptor almost always expressed in a quarter of neurons labeled by TRPV1 in our preliminary study, intratracheal instillation of DiI was subsequently performed in additional four animals to trace pulmonary neurons in the ganglia and double-labeling of DiI+EP3 was examined in these neurons.

#### Series III—To determine the electrophysiological characteristics of PGE_2_- and CAP-induced currents in vagal pulmonary C-neurons and define the roles of TRPV1 and EP3 receptor in these currents

The nodose/jugular ganglia of guinea pigs (n = 15) pretreated with DiI were extracted, primary neurons were cultured overnight, and the currents triggered by PGE_2_- or CAP were recorded on neurons labeled with DiI and a cell size less than 20 μm by using the whole-cell patch clamp technique. PGE_2_ or CAP was ejected onto the target neuron via a pipette (~15 μm away) driven by air pressure in the following six sets of the experiment: 1) and 2) the currents induced by CAP (1.5 μM) [38, 41] and PGE_2_ (up to 20 μM) [42] were recorded respectively; 3) and 4) CAP-induced currents were recorded with application of JNJ 17203212 (20 μM) or L-798106 (6 μM) [40, 43] in the bath solution 30 min prior to CAP; and 5) and 6) the same protocols mentioned in 3) and 4) were carried out with the exception that PGE_2_-, but not CAP-, currents were recorded.

#### Series IV—To electrophysiologically test the effects of PGE_2_ on CAP-currents and CAP on PGE_2_-currents as well as the contributions of TRPV1 and EP3 receptor to both currents

The preparation and recording on nodose/jugular ganglionic neurons of guinea pigs (n = 15) were the same as that described above with the exception that PGE_2_ (20 μM) and CAP (1.5 μM) were sequentially ejected via two separate adjacent pipettes driven by air pressure (see Fig 6A). The currents induced by ejecting CAP followed by ejecting PGE_2_ with an interval of 30 s were recorded without or with application of JNJ 17203212 (20 μM, for the first and second set) in bath solution 30 min prior to the stimulations. The same protocols were performed in the third and fourth set with two exceptions that the order of the sequential stimulation was reversed as PGE_2_ flowed by CAP and that L-798106 instead of JNJ 17203212 was employed in bath solution. The data of PGE_2_- and CAP-induced currents without pretreatment from *Series III* were utilized as Ctrl in this study.

Our pilot experiment showed that PGE_2_ facilitated CAP-induced currents in vagal pulmonary C-neurons. Because the PKA signaling pathway is reportedly involved in EP2 receptor-mediated facilitation of vagal pulmonary C-neural response to CAP in rats [29, 30], we tested if the facilitating effects of PGE_2_ on CAP-currents via EP3 receptor in guinea pigs was also dependent on PKA. To this end, the currents in response to PGE_2_ and CAP alone and to CAP coupled with PGE_2_ pretreatment as mentioned above were recorded before and after application of H-89 (an inhibitor of PKA, 20 μM) in the bath solution (n = 10).

Our preliminary data showed an inhibition of PGE_2_-induced currents after CAP pretreatment (30 s after a previous CAP ejection) and an aggravation of this inhibition after adding JNJ 17203212 in bath solution. This finding raised an interesting question as to how the CAP pretreatment suppressed PGE_2_-induced currents. To address this issue, we tested if there was a desensitization of TRPV1 induced by CAP pretreatment, contributing to the suppression of the subsequent PGE_2_ stimuli as PGE_2_-induced currents are dependent on TRPV1 activation. To this end, we compared the neural responses to the first and second ejecting CAP with the same interval of 30 s in additional vagal pulmonary C-neurons.

### Aerosol exposure to CA and PGE_2_

The plethysmograph chamber was continuously flushed with normoxic gas mixtures (2 L min^-1^) mingled with or without a given nebulized aqueous solution. The solutions containing CA, PGE_2_, and the antagonists were aerosolized by using the nebulizer. The delivery time for CA and PGE2 was 10 min, while that for vehicle, JNJ 17203212, or L-798106 was 5 min. The output rate of delivered aerosol was 0.5 ml min^-1^ with a volume median diameter of 2.5–4.0 μm (per the manufacturer’s indications). The plethysmograph chamber was placed in a standard exhaust fume hood (size: 3 x 6 ft).

### Primary neuron culture

The ganglionic neurons were cultured in the same as the previous report [38]. Briefly, after euthanasia, the ganglia underwent quick extraction and were then cut into pieces and placed in 0.15% type IV collagenase to incubate for 120 min in 5% CO_2_ in air at 37°C. The ganglia suspension was centrifuged (150 g, 5 min) and supernatant aspirated. The cell pellet was resuspended in 0.10% trypsin for 1 min and centrifuged (150 g, 5 min); the pellet was then resuspended in a modified DMEM/F12 solution (supplemented with 10% heat-inactivated fetal bovine serum, 100 units/ml penicillin, 100 μg/ml streptomycin, and 100 μM minimum essential media nonessential amino acids) and gently triturated with a series of small-bore fire-polished Pasteur pipettes. Myelin debris was separated and discarded after centrifugation of the dispersed cell suspension (500 g, 8 min) through a layer of 15% bovine serum albumin. The cell pellet was resuspended in the modified DMEM/F12 solution, plated onto poly-L-lysine-coated glass coverslips, and incubated overnight (5% CO_2_ in air at 37°C).

### Experimental endpoints

#### Cough

As we previously reported [39, 44], three lines of signals including animal body posture (video), cough sound (audio), and respiratory flow were monitored, recorded, and analyzed to define coughs. An electret condenser lavalier microphone (model ECM-V1BMP, sensitivity: 43 dB, Sony, Japan) system was mounted in the roof of the chamber to record sound with the manufacturer’s technical parameters to determine the sound intensity. A video camera (Microsoft LifeCam Studio) was placed outside of the chamber to monitor animal body posture. A Buxco pneumotachograph (pressure transducer) was attached to the chamber for recording the respiratory flow. Both the sound and flow signals were collected, digitized at a sampling rate of 20 kHz via a PowerLab unit (model 8/35, ADInstruments Inc., Colorado Springs, CO) and recorded continuously to computer files using a Dell desktop computer (XPS 8930, Dell, Round Rock, Texas) with LabChart 8.0 Pro software (ADInstruments Inc.). Cough response was defined by the simultaneous appearance of three characteristics: 1) a transient and great change in the airflow (a deep inspiration coupled with a big expiration); 2) a typical cough sound with the peak power density at ~1.0 kHz of frequency spectrum (sneeze at 3.5–6.5 kHz); and 3) animal body posture (forward stretching of the neck and evident abdominal movement). All signals were monitored and recorded continuously 5 min before and 15 min during aerosol delivery (10 min for CA or PGE_2_ plus 5 min for the antagonist) and 30 min post-delivery.

#### Immunohistofluorescence

After euthanasia, the guinea pig was perfused transcardially with 30 ml of saline followed by 200 ml of 4% paraformaldehyde in 0.1 M PBS (pH 7.4). The bilateral nodose ganglia were removed and post-fixed for 2 h in the same fixative, rinsed with PBS, overnighted in 30% sucrose in 0.1 M PBS, and frozen in a Tissue-Tek OCT embedding medium (Miles; Elkhart, IN). The nodose/jugular ganglia was serially sectioned (10-μm-thick sections) by a precision cryostat, slices were mounted on the gelatin chromalum-coated slides, and the slides were stored at -70°C until they were processed for immunohistofluorescence studies [38]. Frozen slides were selected, dried at room temperature for 10 min and washed three times in PBS; these were incubated for 4h with blocking solution (0.1% TritonX-100 containing 10% normal goat serum in PBS). The slices of bilateral nodose/jugular ganglia were incubated overnight at 4°C with primary antibody rabbit anti-EP3 (1:100; Abcam, ab189122) and mouse anti-TRPV1 (1:300; Abcam, ab203103) diluted in the blocking solution. The sections were then washed three times in PBS and incubated for 4 h at room temperature with second antibody goat anti-rabbit IgG conjugated with Alexa-488 fluorochrome and goat-anti-mouse Alexa-593 fluorochrome (1:200 dilutions, Invitrogen, CA). For a DiI pretreatment slice, only EP3 antibody was used. Lastly, the slices were coverslipped with antifade reagent (Life Technologies Co. USA). Digital micrographs of DiI, TRPV1 and EP3 receptor immunoreactivity (IR) were acquired using a 10X objective with a digital camera (AxioCam HRm, Zeiss, Germany) connected to an epifluorescence microscope (Axioplan 2 FS, Zeiss, Germany). The grayscale values of TRPV1-IR and EP3-IR were measured in vagal pulmonary C-neurons with Image J (1.48v) software, respectively. A representative slice with the largest area from each nodose ganglion was used for cell count. The populations of the neurons single-labeled by DiI and double-labeled by DiI+EP3 or TRPV1+EP3 were calculated.

#### Patch clamp recording

The whole-cell voltage patch clamp recording technique was similar to those described previously [38]. All recordings were made on the neurons cultured on coverslips at a V_H_ of -70 mV. Neurons were superfused (2 ml/min) continuously with standard extracellular solution (bath solution) containing the following chemicals (in mM): 136 NaCl, 5.4 KCl, 1.8 CaCl_2_, 1 MgCl_2_, 0.33 NaH_2_PO_4_, 10 glucose, and 10 HEPES; pH was adjusted to 7.4 with NaOH. In some cases, JNJ 17203212 (20 μM) or L-798106 (6 μM) were added into the bath solution. Whole cell patch clamp was performed by using Axopatch 200B, Digidata 1440A, and pClamp 10.5 software (Molecular Devices, Palo Alto, CA). Only vagal pulmonary C-neurons were investigated in the study and identified by retrograde labeling and cell size (< 20 μm) [35] with fluorescence microscopy. Patch pipettes were pulled from borosilicate glass capillary tubings (G-1.5, Narishige) with a Narishige PC-10 two-stage electrode puller (Narishige International Inc., NY, USA). The patch pipette solution had the following composition (in mM): 92 potassium gluconate, 40 KCl, 8 NaCl, 1 CaCl_2_, 0.5 MgCl_2_, 10 EGTA, and 10 HEPES; pH was adjusted to 7.2 with KOH. The pipette resistance was 3–5 MΩ when filled with the above saline. The PGE_2_ and CAP were applied via two separate pipettes (diameter: 10 μm) manipulated by a pressure-driven micro-injection system (Picospritzer II, General Valve Corporation, Fairfield, NJ) with consistent parameters (10 s, 2 psi), and the volume was 1.2 to 1.6 nl for each application. The signals were filtered at 2 kHz and sampled at 10 kHz. Series resistance (6 ~18 MΩ) was monitored throughout the recordings and data were discarded if the resistance changed by >20%. The neurons lacking the response to CAP or PGE_2_ were not countered in this study. The experiments were performed at room temperature (~22°C).

### Animal euthanasia

After completion of the experiment, the animals were euthanized by receiving 1:4 diluted euthasol at 150–200 mg/kg minimum by intraperitoneal injection and euthanasia was confirmed by pneumothorax (SOP ACS-0334-Euthanasia of Small Animals).

### Reagents

All chemicals were purchased from Sigma-Aldrich (St. Louis, MO) or Tocris Bioscience (Minneapolis, MN) unless otherwise stated. A stock solution of capsaicin (1 mg/ml) was prepared in 10% Tween 80, 10% ethanol, and 80% saline, and then diluted in standard extracellular solution to the final concentration before the experiments. PGE_2_, TRPV1 antagonists (JNJ 17203212) and EP3 receptor antagonist (L-798106) were prepared in 1% DMSO and then diluted in saline or extracellular solution for each time. CA and H-89 was prepared with saline directly.

### Data acquisition and statistical analysis

Cough (bout of coughing) numbers *in vivo* and the amplitude, rising and decay time of the evoked currents *in vitro* were expressed as absolute values. The ratio of single-labeled vs. double-labeled neural population was expressed as percentage. Group data were reported as mean and standard error (means ± SE). The Kolmogorov-Smirnov test was conducted to confirm the normal distribution of the data groups. The variables were compared between CA or PGE_2_ exposures with and without saline, JNJ 17203212, or L-798106 as well as H-89 by using Two-way ANOVA. The data of immunoreactivities (ratio) and patch clamp were analyzed by using Student t-test or one-way ANOVA as well as Tukey Test (*P*-values < 0.05 were considered significant).

## Results

### The patterns of the cough responses to CA and PGE_2_ were markedly different

**Fig 1:**
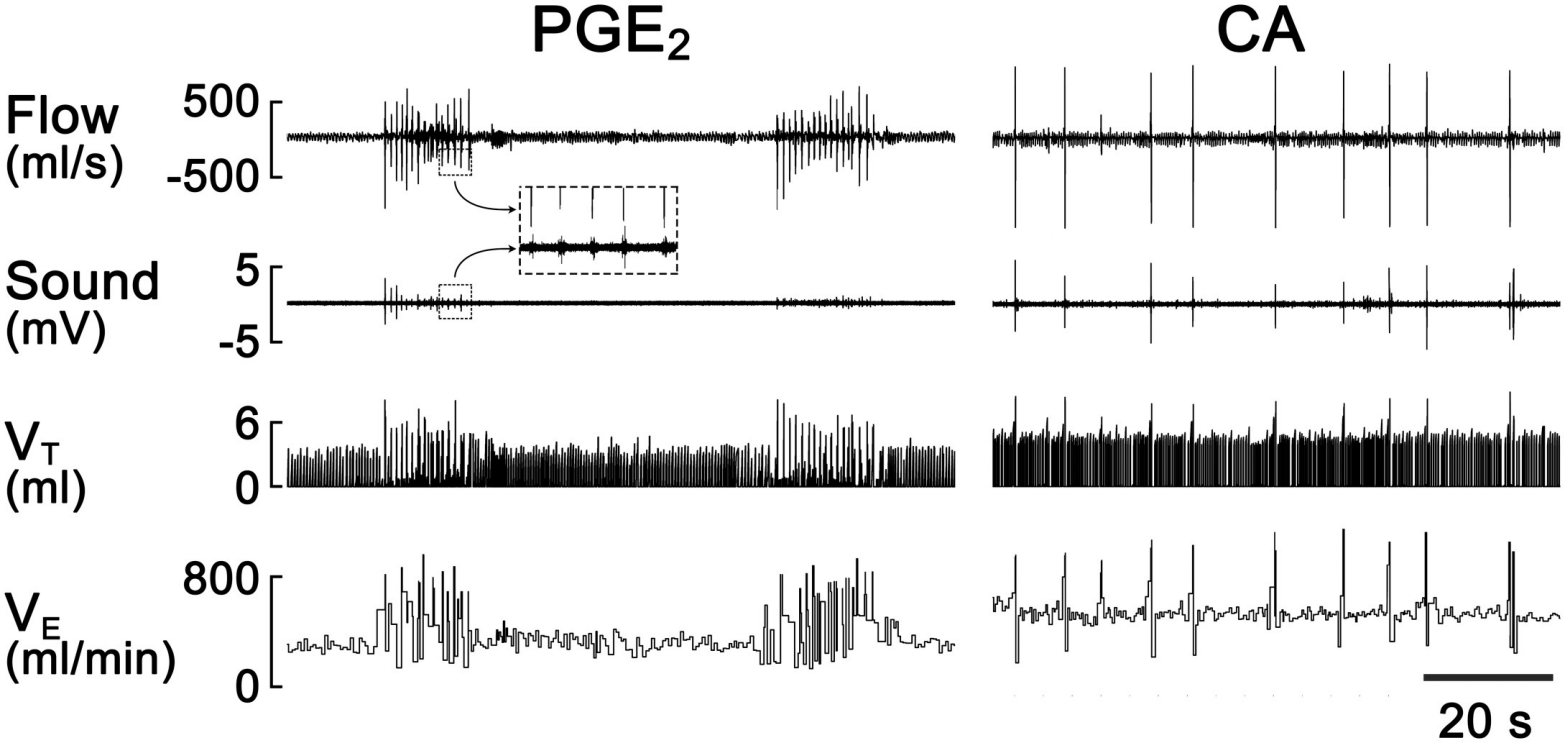
Comparison of the typical cough responses to PGE_2_ and CA in two unanesthetized guinea pigs respectively. The traces from top to bottom are air flow, sound, tidal volume (V_T_), and minute ventilation (V_E_). The insert of a dash-lined box shows the enlarged flow and sound signals.

Cough responses to CAP and PGE_2_ possess distinct patterns according to cough latency, rate (coughs/min), sound duration, intensity and animal body posture [39]. As shown in Fig 1, inhalation of aerosolized PGE_2_ (0.43 mM) or CA (150 mM) for 10 min evoked different cough patterns. The former is bout(s) of coughing (absent or very weak cough sound) with evident abdominal movement, while the latter is individual and loud coughs associated with forward stretching of the neck. In addition, the change in respiratory flow is greater in the CA-evoked than the PGE_2_-evoked coughs, consistent with the previous studies in humans and unanesthetized guinea pigs [14, 15, 45–50].

### PGE_2_-evoked cough was inhibited by TRPV1 or EP3 receptor antagonist but CA evoked-cough was only blunted by TRPV1 antagonist

**Fig 2:**
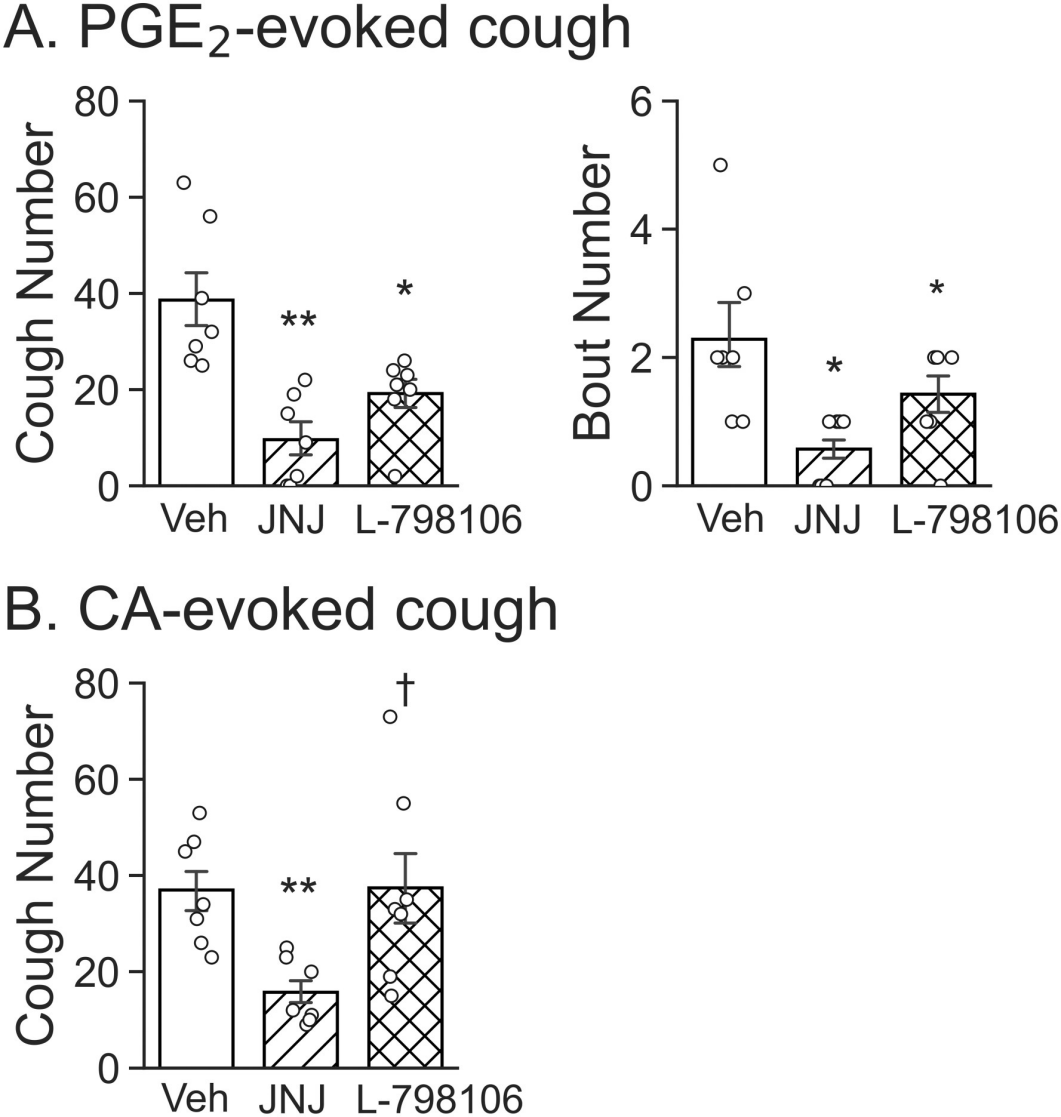
Effects of inhalation of vehicle (Veh), TRPV1 antagonist (JNJ 17203212, JNJ) or EP3 receptor antagonist (L-798106) on the PGE_2_-evoked (A) and CA-induced cough (B). n = 7/subgroup; * P < 0.05 and ** P < 0.01 as compared to Veh. † P < 0.05 and ‡ P < 0.01, L-798106 vs. JNJ 17203212 pretreatment.

This study aimed to test the dependency of the cough response to PGE_2_ or CA on TRPV1 and EP3 receptor. The guinea pigs, following 5 min exposure to vehicle, JNJ 17203212 (1.5 mg/ml) or L-798106 (1.5 mg/ml), were exposed to aerosolized PGE_2_ or CA for 10 min (6 groups). As presented in Fig 2A, inhalation of aerosolized PGE_2_ (0.43 mM) evoked 2.29 ± 0.56 bouts of coughing that contained 38.57 ± 6.19 cough numbers. PGE_2_-evoked coughs were decreased by 75% after JNJ 17203212 (9.57 ± 3.78) and by 50% after L-798106 (19.14 ± 3.27). Similarly, PGE_2_-evoked bouts of coughing were strikingly attenuated by 75% and 37% after JNJ 17203212 (0.57 ± 0.22) and L-798106 (1.43 ± 0.32) respectively. As illustrated in Fig 2B, CA evoked 37.0 ± 4.66 coughs after vehicle and this response was reduced by 58% (15.71 ± 2.75) after JNJ 17203212. In sharp contrast, L-798106 did not affect CA-induced cough. In additional two animals, the CA-evoked coughs (35.3 ± 3.51) were not affected by triple-dosed L-798106 (4.5 mg/ml; 36.0 ± 3.22). It should be noted that inhalation of vehicle, JNJ 17203212, and L-798106 for 5 min per se did not significantly change baseline ventilation (Table 1).

**Table 1:** Baseline ventilation before and after pre-inhalation of JNJ 17203212 or L-798106.

	V_T_	f_R_	V_E_
	(ml/s)	(breaths/min)	(ml/min)
**Vehicle**	3.00 ± 0.07	118.8 ± 9.2	348.2 ± 26.3
**JNJ 17203212**	3.04 ± 0.24	108.2 ± 5.7	322.8 ± 21.6
**L-798106**	3.06 ± 0.11	106.3 ± 3.3	343.1 ± 26.0

The ventilations were not changed before (vehicle) and after JNJ 17203212 or L-798106 inhalation. V_T_, Tidal volume; f_R_, Respiratory frequency; V_E_, Minute ventilatory volume. n = 14/group.

### EP3-labeled vagal pulmonary sensory neurons co-expressed TRPV1

**Fig 3:**
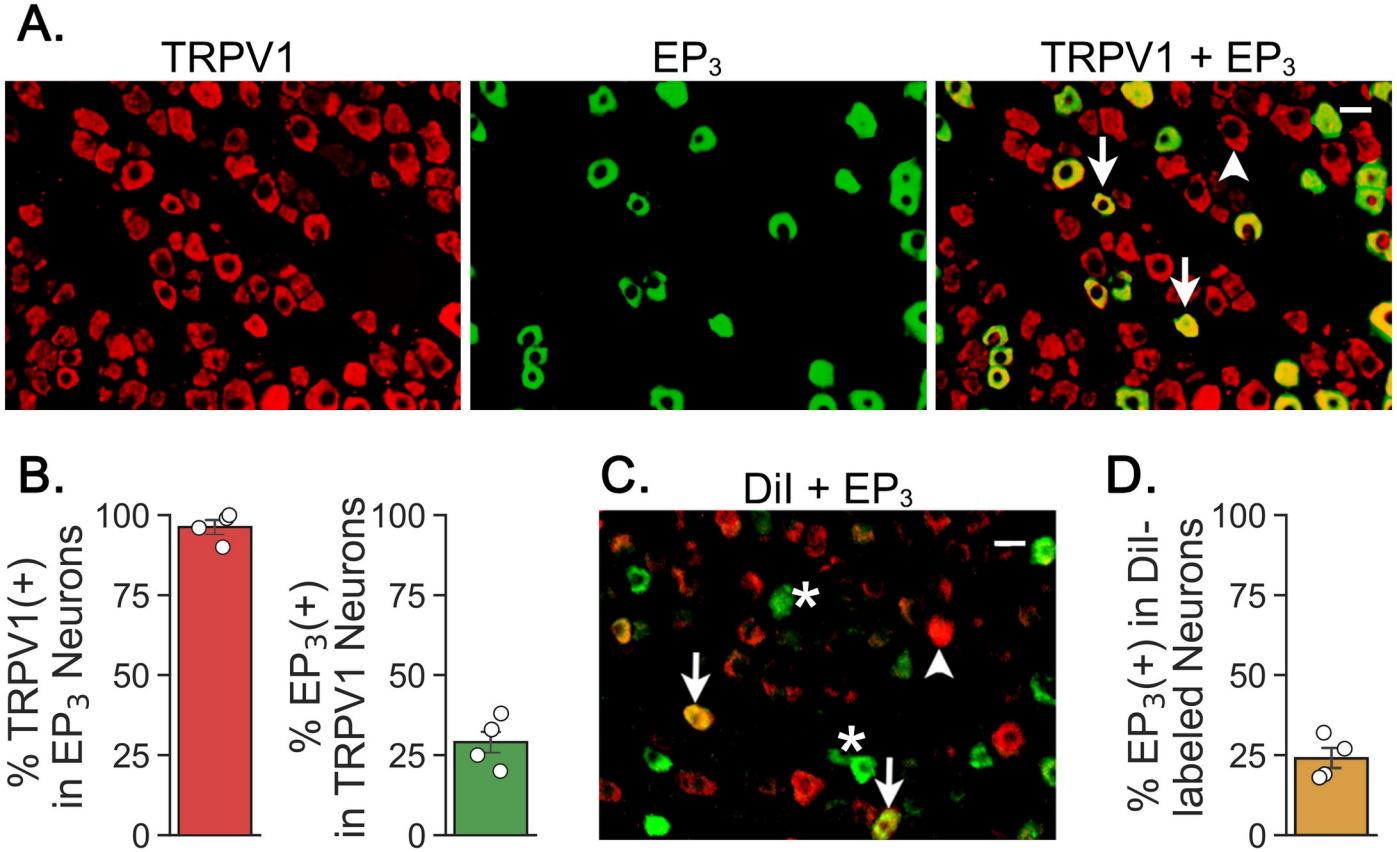
Co-expression of TRPV1 and EP3 or DiI and EP3 in nodose ganglion neurons. A: Nodose ganglionic neurons single-labeled with TRPV1 (left) or EP3 (middle) and double-labeled with TRPV1+EP3 (right). Arrows and arrowhead point to the double- and single-labeled neuron(s) respectively. Bar = 25 μm. B: The corresponding group data show that almost all EP3-labeled neurons co-express TRPV1 and 31% of TRPV1 neurons are co-labeled with EP3 (n = 4). C: Nodose ganglionic neurons single-labeled with EP3 or DiI (green or red, as marked by “*” or arrowhead) and double-labeled with EP3+DiI as pointed by arrows. D: Group data showing that 24% of DiI-labeled neurons are co-labeled with EP3 (n = 4).

By using an immunohisto-fluorescence approach, the expression of both TRPV1 and EP3 receptor presented in nodose and jugular ganglion neurons was first identified in this study. We found that TRPV1 was expressed in 80% ± 12% of neurons in the nodose ganglion and 64% ± 16% neurons in the jugular ganglion and approximately 1/3 of these TRPV1-labeled nodose and jugular neurons co-expressed EP3 with the cell size smaller than 20 μm (Fig 3A and 3B). Because EP3 receptors always co-expressed TRPV1 in vagal sensory C-neurons, to what extent EP3 had expressed in vagal pulmonary C-neurons was subsequently examined. Approximately 24% of the DiI-labeled neurons co-expressed EP3 and some DiI-unstained neurons also expressed EP3; again, these co-labeled neurons had cell size smaller than 20 μm (Fig 3C and 3D). Because DiI-unstained neurons could be non-pulmonary or pulmonary neurons unlabeled by DiI (due to the technical limitation), no attempt was made in this study to analyze EP3 expression in these neurons.

### PGE_2_ and CAP triggered currents in the pulmonary C-neurons

**Fig 4:**
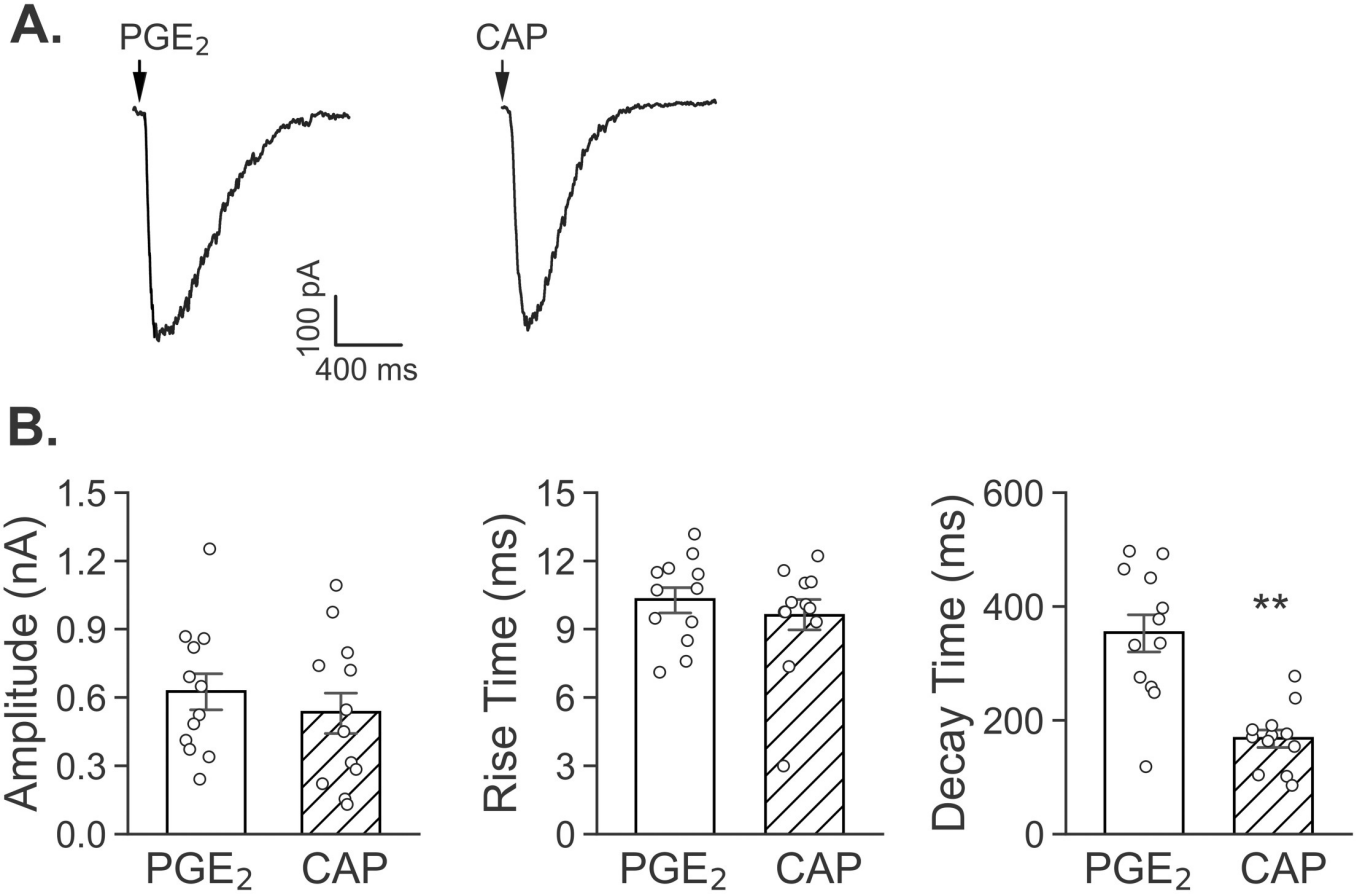
Kinetic characteristics of CAP- and PGE_2_-induced currents in two groups of vagal pulmonary C-neurons. The amplitudes and 10–90% rise times did not have a significant difference between these two currents, while the decay time of PGE_2_-induced currents were 2-fold longer than that of CAP-induced currents. n = 12 neurons in the two groups respectively. In our patch clamp studies, “n” represents numbers of the neurons tested. ** P < 0.01, compared to CAP-induced currents.

In order to test if PGE_2_ could trigger currents in the vagal pulmonary C-neuron that were identified by DiI labeling and a cell size < 20 μm with membrane capacitance (C_m_) were around 17.98 ± 0.56 pF, we initially ejected different concentrations of PGE_2_ onto the recorded neurons. The ejecting concentration of PGE_2_ when lower than 10 μM rarely triggered the currents (n = 8), while PGE_2_ concentration at 10 μM and 20 μM evoked the currents in around 50% and 85% of the neurons tested (n = 10 and 14). We also recorded the CAP-induced currents on the vagal pulmonary C-neurons (n = 12) and compared the kinetic characteristics of the currents in response to CAP (1.5 μM) and PGE_2_ (20 μM), including amplitude, rise time and decay time. The results showed that the amplitude and 10–90% rise time of both currents were not found to have a significant difference, but the 37% decay time of PGE_2_-induced currents was longer than that of CAP-induced currents significantly (Fig 4).

### PGE_2_-induced currents were inhibited by EP3 receptor or TRPV1 antagonist while CAP-induced currents were only suppressed by TRPV1 antagonist

**Fig 5:**
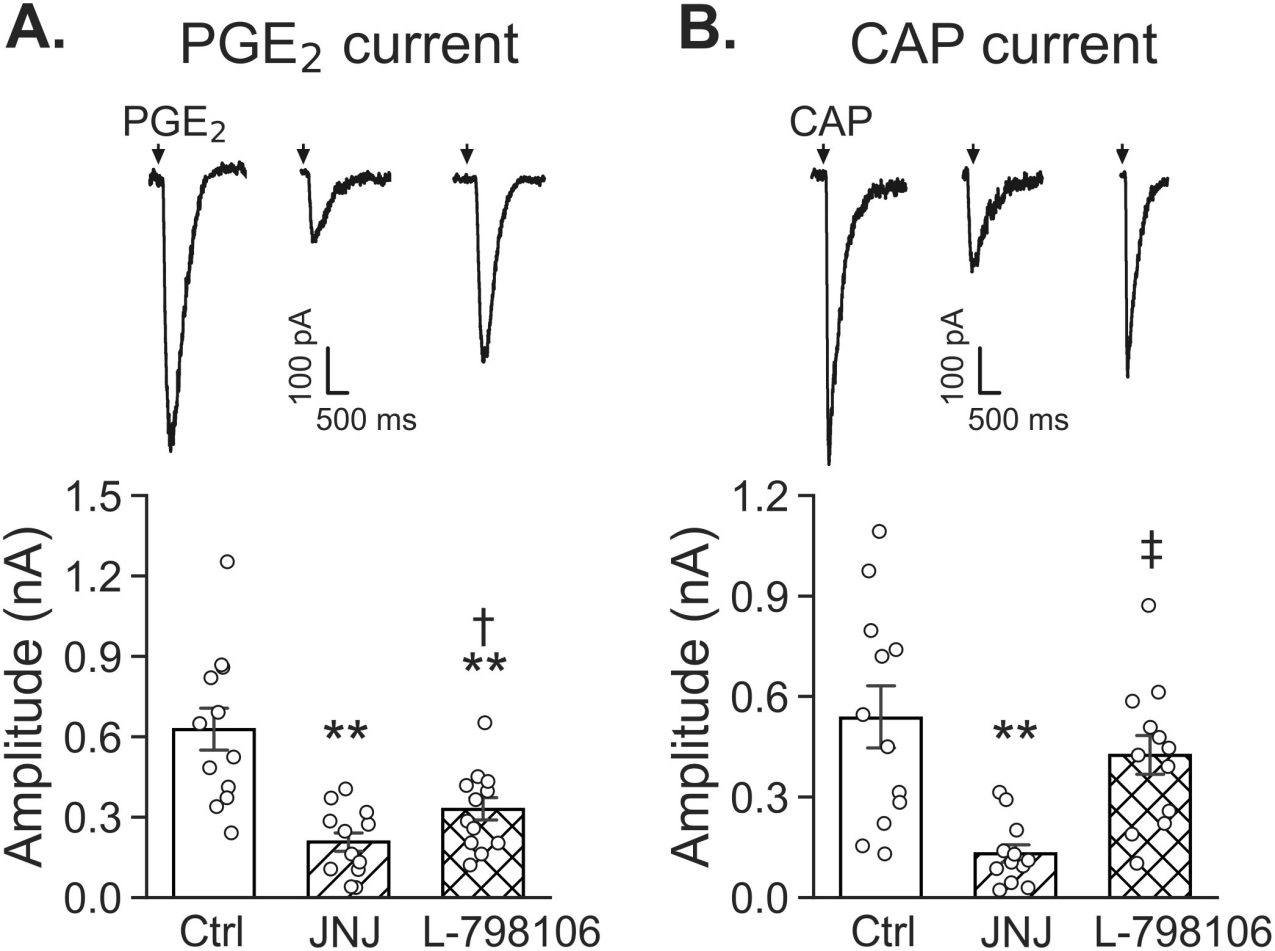
The effects of JNJ 17203212 (JNJ) and L-798106 on the PGE_2_- (A) and CAP-induced currents (B) respectively. The amplitudes of PGE_2_-induced currents are inhibited by both JNJ 17203212 and L-798106, but those of CAP-induced currents are suppressed by JNJ 17203212 and not by L-798106. JNJ 17203212 (20 μM) or L-798106 (6 μM) is applied into the bath solution 30 min ahead of recording (n = 12 for each group). * P < 0.05 and ** P < 0.01 as compared to Ctrl. † P < 0.05 and ‡ P < 0.01, L-798106 vs. JNJ 17203212 pretreatment.

To examine if both TRPV1 and EP3 receptor contribute to PGE_2_- and CAP-induced currents, we applied TRPV1 antagonist JNJ 17203212 (20 μM) or EP3 receptor antagonist L-798106 (6 μM) in bath solution 30 min before the recording. As shown in Fig 5A, compared to the amplitudes of PGE_2_-induced currents in Ctrl (data from Fig 4A), these values were reduced by 67% in the neurons exposed to JNJ 17203212 and by 47% in the neurons exposed to L-798106. Moreover, the amplitudes of CAP-induced currents in Ctrl (data from Fig 4A) were markedly dropped by 74% after JNJ 17203212, but not significantly altered by L-798106. Interestingly, these antagonists failed to significantly alter the rise time and decay time in response to PGE_2_ and CAP. The PGE_2_ and CAP-evoked rise time (ms) are 10.30 ± 0.55 and 9.61 ± 0.70 before and 11.61 ± 0.46 and 10.43 ± 0.59 after application of JNJ 17203212, and 10.21 ± 0.74 and 10.06 ± 0.47 before and 11.06 ± 0.82 and 9.82 ± 0.65 after application of L-798106. The decay times of these currents are also not changed. These data indicate that TRPV1 is involved in PGE_2_-induced currents, but EP3 receptor is not involved in generation of CAP-induced currents.

### PGE_2_ pretreatment facilitated CAP-induced currents through EP3 receptors, while CAP pretreatment depressed PGE_2_-induced currents

**Fig 6:**
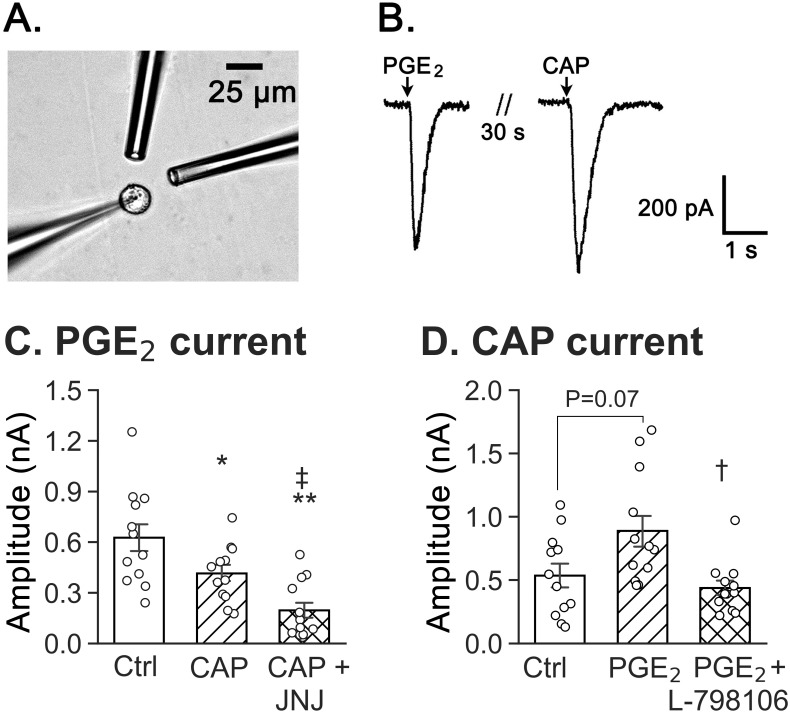
Cross-effect between CAP and PGE_2_ on the evoked currents of vagal pulmonary C-neurons. A: A recording electrode on a vagal pulmonary C-neuron retrogradely traced by DiI is surrounded by two adjacent pipettes for drug delivery with ~15 μm distance to the neurons. B: PGE_2_- and CAP-induced currents in a vagal pulmonary C-neuron with an interval of 30 s between the stimulations. C: The PGE_2_-induced currents are inhibited by ~33% after CAP-pretreatment alone and by ~69% after CAP-pretreatment coupled with TRPV1 antagonist. D: PGE_2_-pretreatment enhances CAP-induced currents via acting EP3 receptor. n = 12/group, * P < 0.05 and ** P < 0.01 as compared to Ctrl. † P < 0.05 and ‡ P < 0.01, CAP + JNJ (JNJ 17203212) vs. CAP pretreatment or PGE_2_ + L-798106 vs. PGE_2_ pretreatment.

In order to verify whether both PGE_2_ and CAP could trigger currents in the same pulmonary C-neurons, we employed an adjacent drug delivery with double-pipettes to eject CAP (1.5 μM) and PGE_2_ (20 μM) separately and sequentially as shown in Fig 6A. PGE_2_ and CAP were ejected onto the given neurons retrogradely traced by DiI with a cell size < 20 μm through a pressure-driven micro-injection system (10 s, 2 psi) with an interval of 30 s. Both PGE_2_- and CAP-induced currents were recordable in some neurons as exhibited in Fig 6B. Of 38 vagal pulmonary C-neurons recorded, 35 neurons showed the response to CAP, 30 neurons response to both CAP and PGE_2_ and only one neuron response to PGE_2_ alone. In other words, 92% (35/38) of the vagal pulmonary neurons recorded in this study are CAP sensitive (i.e., vagal pulmonary C-neurons) and 85% (30/35) of the latter are also sensitive to PGE_2_, while only 3% (1/38) of neurons are exclusively responsive to PGE_2_.

To clarify whether PGE_2_ facilitated CAP-induced currents via acting on EP3 receptors and whether CAP affected PGE_2_-induced currents via acting on TRPV1, the sequential ejections (CAP followed by PGE_2_ or PGE_2_ followed by CAP) were performed in four groups of vagal pulmonary C-neurons without or with application of JNJ 17203212 (20 μM) or L-798106 (6 μM) in bath solution 30 min prior to the stimulations. We found that the amplitudes of PGE_2_-induced currents (Ctrl data from Fig 4A) were significantly decreased by 33% after CAP pre-ejecting, and by 69% after CAP pre-ejecting with JNJ 17203212 in bath solution (Fig 6C). In sharp contrast, the amplitudes of CAP-induced currents (Ctrl data from Fig 4A) were significantly increased by 67% after PGE_2_ pre-ejecting and this increase was abolished with a bath solution containing L-798106 (Fig 6D). This result indicates that PGE_2_ facilitates CAP-induced currents fully dependent on EP3 receptor in our setting of drug application.

### PKA inhibitor almost blocked PGE_2_-current and the facilitating effect of PGE_2_ on CAP-induced currents

**Fig 7:**
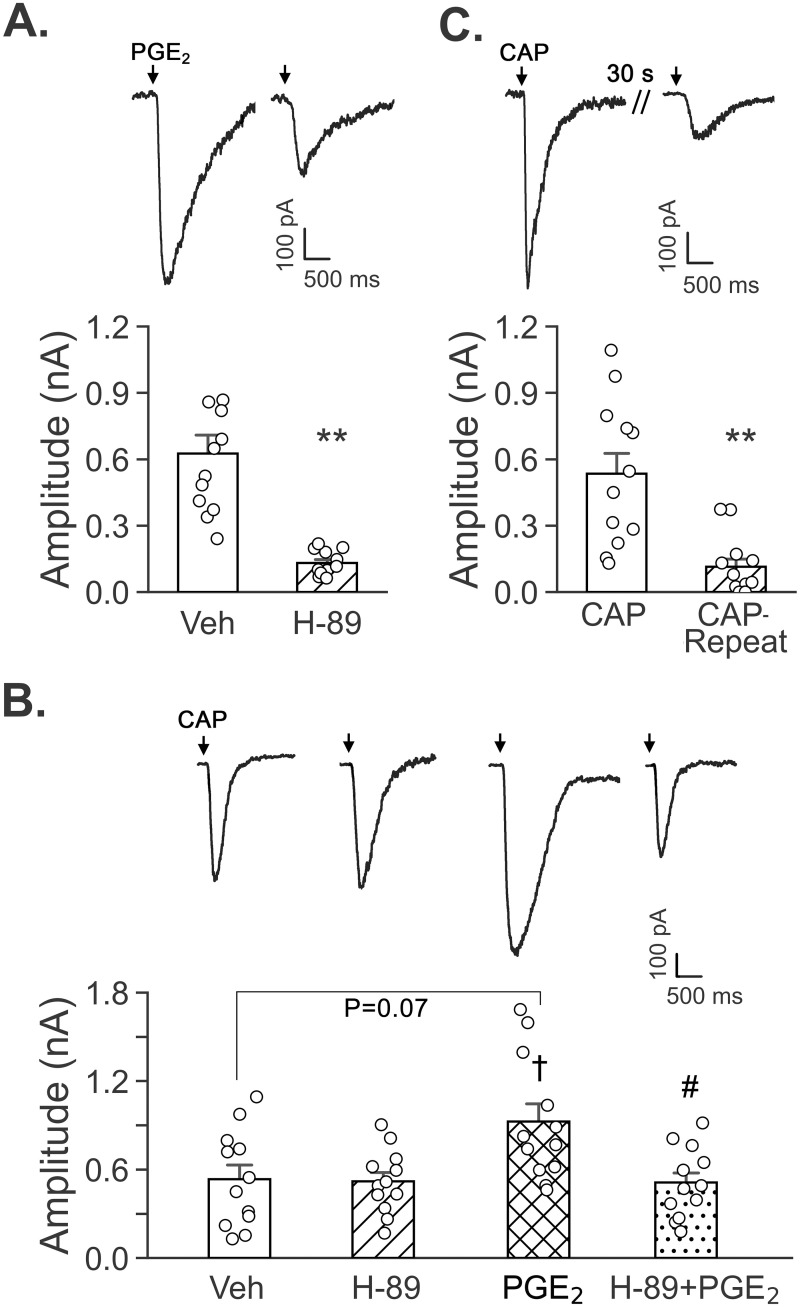
Effects of H-89 on PGE_2_-, CAP-, and PGE_2_+CAP-evoked currents and the desensitization of CAP-evoked currents by the first CAP in vagal pulmonary C-neurons. Typical recordings and the corresponding group data to show the impacts of H-89 (a PKA inhibitor) on the evoked currents in response to PGE_2_ (A, n = 12), and CAP alone or CAP with PGE_2_ pretreatment (B, n = 12). ** P < 0.01 as compared to the data without H-89, † and # P < 0.05, as compared to CAP alone (with Veh pretreatment) and PGE_2_ respectively. C: Typical recordings to show the current responses to the first and second CAP in a vagal pulmonary C-neuron and the corresponding group data (n = 12). Compared to the first CAP ejection, the amplitudes of the currents evoked by the second ejecting CAP (CAP-Repeated) were markedly reduced. ** P < 0.001 as compared to the first CAP ejection.

To test the role of the PKA signal pathway in the facilitating effect of PGE_2_ on CAP-induced currents, we compared the evoked currents in response to PGE_2_ and CAP alone and to CAP coupled with PGE_2_ pretreatment with and without application of H-89 (an inhibitor of PKA, 20 μM) in the bath solution. We found that H-89 almost blocked the PGE_2_-induced currents and its augmenting effect on CAP-induced currents, while it did not significantly affect CAP-induced currents without PGE_2_ pretreatment (Fig 7A and 7B).

### Pre-ejection of CAP significantly desensitized CAP-induced current

As mentioned above, we found that the amplitudes of PGE_2_-induced currents were decreased by 33% 30 s after ejecting CAP. Because PGE_2_-induced currents are largely dependent on TRPV1 activation (Fig 5A), we tested if there was a desensitization of TRPV1 following the CAP ejection that contributed to reduction of the amplitudes of currents induced by subsequent ejection of PGE_2_. To this end, CAP was applied twice in the same vagal pulmonary C-neurons with the same interval (30 s) as mentioned above. Compared to the first CAP ejection, the current responses evoked by the second CAP ejection were blunted by 78% (Fig 7C), clearly indicating a desensitization of TRPV1 30 s after CAP ejection, consistent with previous reports [51, 52].

## Discussion

The present studies from *in vivo* to *in vitro* revealed several new findings. (1) PGE_2_-evoked cough was suppressed by inhalation of aerosolized TRPV1 or EP3 receptor antagonist, while CA-evoked cough was only inhibited by TRPV1 antagonist. (2) Approximately 24% vagal pulmonary C-neurons co-expressed TRPV1 and EP3 with a cell size often smaller than 20 μm. (3) PGE_2_ at 20 μM could trigger immediate inward currents in vagal pulmonary C-neurons. (4) PGE_2_-induced currents were inhibited by both EP3 receptor and TRPV1 antagonists, but CAP-induced currents were uniquely suppressed by TRPV1 antagonists. (5) Pre-ejection of PGE_2_ acting on EP3 receptor facilitated CAP-induced currents through PKA signaling pathway, while PGE_2_-induced currents were inhibited by pre-ejection of CAP (desensitization). Taken together, our results suggest that both EP3 and TRPV1 receptors are required for full expression of PGE_2_-induced vagal pulmonary C-neuronal excitation *in vitro* and cough *in vivo*, while EP3 receptors are not involved in triggering CAP-induced neuronal currents and cough in guinea pigs.

Studies have shown that PGE_2_-induced coughs are largely attenuated by IP injection of JNJ 17203212 (100 mg/kg) or L-826266 (300 mg/kg) in unanesthetized guinea pigs [14, 26]. CAP-induced coughs are also suppressed and eliminated by IP injection of JNJ 17203212 at lower (10–30 mg/kg) and higher doses (> 100 mg/kg) respectively [26], but no study has been carried out to test the effect of EP3 receptor antagonist on CAP-induced cough. To relatively focus on the airways, we tested if TRPV1 or EP3 receptor antagonist administrated via aerosol inhalation was sufficient to suppress CA- and/or PGE_2_-induced coughs. We found that PGE_2_-induced coughs were significantly decreased by 75% and 50% after inhaling JNJ 17203212 and L-798106 respectively, and CA-evoked coughs were reduced by 58% after JNJ 17203212 and unchanged after L-798106 application. This finding suggests that activation of airway TRPV1 and EP3 receptor is essential for fully expressing PGE_2_-induced cough. The fact that inhaling L-798106 fails to affect CA-induced cough reveals, for the first time, that EP3 receptors under pathogen free condition has a limited contribution to this type of cough. There are several advances in inhalation approach used in this study. First, compared to IP injection of JNJ 17203212 and L-826266 (30 and 300 mg/kg) [14, 26], inhalation of the same antagonists at much lower doses (3.75 mg/2.5 ml for both JNJ 17203212 and L-798106) produces similar antitussive effects. Second, the post-administration duration required to produce the antitussive effect is shorter through inhalation than that via IP injection (5 min vs. 40 min). Third, owing to the presence of TRPV1 and EP3 receptor in a variety of organs, such as the brains (for a review, see [53, 54]), inhalation of these antagonists should have less possible side effects as compared to systemic administration. Collectively, our results showing the higher antitussive efficacy of these antagonists via inhalation than systemic administration support that PGE_2_-evoked cough requires both TRPV1 and EP3 receptor activation of airway sensory fibers, while CA-evoked cough depends the former but not the latter.

PGE_2_-induced depolarization of the isolated vagal nerve from human, guinea pig, and mouse is reportedly reduced by local application of JNJ 17203212 or L-826266 *in vitro* [14, 26]. However, the vagal nerve contains sensory fibers innervate airways/lungs and other visceral organs. The expression of TRPV1 has been identified in vagal pulmonary C-neurons [34, 35] and that of EP3 receptors in the nodose ganglion and dorsal root ganglion neurons [36, 37, 55]. In this study, we confirmed that 24% of vagal pulmonary C-neurons express EP3 receptor with cell size < 20 μm (Fig 3). In agreement, mRNA expression of EP3 has been identified in rat nodose ganglionic neurons with small cell size [36, 56]. Our morphological data showing the co-expression of TRPV1 and EP3 receptor in vagal pulmonary C-neurons provide a strong rationale for our following electrophysiological studies.

CA/CAP and PGE_2_ are capable of stimulating PCFs respectively [9, 57–60]. However, it is unclear whether the same individual of vagal pulmonary C-neurons is responsive to both CAP and PGE_2_, and if so, whether there is a cross-effect of TRPV1 and EP3 receptor on the excitation of these neurons. In this study, ~50% of vagal pulmonary C-neurons are sensitive to PGE_2_ at 10 μM. Different from our data, PGE_2_ (up to 10 μM) itself failed to evoke any response in afferent sensory neurons innervating rat intestinal wall, but enhanced the serotonin (5-HT)-evoked currents [42]. This discrepancy may be due to the different sensory neurons tested (gastrointestinal vs. pulmonary) and lower concentration of PGE_2_ used (10 vs. 20 μM) compared to our study. Previous studies have shown that 1 μM PGE_2_ causes hyperexcitability in vagal pulmonary C-neurons via acting on EP2 receptor in rats [61, 62]. This, along with our data mentioned above, indicates that PGE_2_ is capable of stimulating both EP2 and EP3 receptors. We did not test if EP2 receptor was also involved in PGE_2_-induced cough *in vivo* and current in vagal pulmonary C-neurons in guinea pigs. However, our data have shown a difference between 24% of vagal pulmonary C-neurons morphologically expressing EP3 and 50–85% of these neurons are functional sensitive to 10–20 μM PGE_2_. This difference (24% vs. 50–85%) supports a possibility that, in addition to EP3 receptors, considerable EP2 receptors exist in vagal pulmonary C-neurons sensitive to PGE_2_ in guinea pigs. We also found that CAP-induced currents were suppressed by 74% after JNJ 17203212 and unchanged by L-798106, while PGE_2_-induced currents were inhibited by 67% and 47% after JNJ 17203212 and L-798106 respectively (Fig 5C and 5D). These cellular data are highly similar to our cough data showing suppression of CAP-induced cough by 58% after inhaling JNJ 17203212 and PGE_2_-induced cough by 75% and 50% after inhalation JNJ 17203212 and L-798106 respectively (Fig 2). There is a similarity of the effects on cough (via inhaling the antagonists mainly acting on the airways) and vagal pulmonary C-neurons (via focal ejecting the same antagonists). This forms a new concept that both TRPV1 and EP3 receptor in vagal pulmonary C-neurons contributes, at least in part, to cough response to PGE_2_.

It is well known that inhalation of CA/CAP provokes individual loud coughs (Type I), while inhalation of PGE_2_ induces bout(s) of smaller and quieter coughs (Type II) in humans and unanesthetized guinea pigs [14, 15, 39, 45–48, 50]. However, the mechanisms underlying the genesis of the two distinct cough patterns remain unexplored. Our morphological and electrophysiological findings mentioned above raise the possibility that the two groups of vagal pulmonary C-neurons expressing TRPV1 alone and TRPV1+EP3 receptor may be accountable for the different cough patterns generated. Although the mechanisms underlying PGE_2_-induced currents via acting EP3 receptor are not clear, we reason that PGE_2_ evokes currents via activating the ion channels including TRPV1. PGE_2_ receptors comprise of four subtypes (EP1- EP4), among which EP3 receptors uniquely couple to Gi protein [63, 64]. Activation of EP3 receptors is reported to decrease the intracellular cAMP concentration and increase the intracellular calcium concentration [32], which are capable of potentiating the activity of TRPV1 [65]. Actually, the importance of the opening of TRPV1 channel in generation of PGE_2_-currents is evident in the present study. Our data demonstrated that PGE_2_-induced currents were largely blunted by blockade or desensitization of TRPV1, strongly suggesting that TRPV1 is a critical component responsible for generating PGE_2_-currents. Because blockade of TRPV1 by JNJ 17203212 largely reduces, but does not eliminate, PGE_2_-induced currents, other ion channels may also be involved in generation of the currents. The lack of effects of L-826266 on CAP-induced currents in our study *in vitro* suggests that the activation of EP3 receptors is not required for CAP-induced currents, consistent with absence of the effect of L-826266 on CAP-induced cough *in vivo*.

Because both TRPV1 and EP3 receptor exist in the same vagal pulmonary C-neuron, we further tested whether CAP- or PGE_2_-induced currents would be facilitated by pre-ejecting PGE_2_ or CAP 30 s ahead. Our results showed that the amplitudes of CAP-induced currents in vagal pulmonary C-neurons were doubled by PGE_2_ (20 μM) pre-ejection and this augmentation was abolished after blockade of EP3 receptor (Fig 6A), consistent with PGE_2_ enhancing the sensitivity of CAP-induced cough [27]. Moreover, this funding indicates that PGE_2_ facilitates CAP-induced currents dependent on EP3 receptor. It was reported that PGE_2_ at 1 μM per se failed to affect neural excitation but enhanced isolated vagal pulmonary C-neural response to CAP in rats via acting on EP2 receptor [29, 30]. PGE_2_ also greatly prolonged the apneic response to CAP in rats [61, 62]. Interestingly, PGE_2_ at 10–20 μM but not < 10 μM induced currents in vagal pulmonary C-neural response in our study. These results offer at least two possibilities to explain why PGE_2_ can evoke either apnea or cough. One is that PGE_2_ at low dose can reflexively produce apnea via acting on EP2 receptor and that at high dose triggers cough via EP3 receptor. Another one is that there are two afferent subsets: the apneic response to PGE_2_ is mediated by the afferents expressing EP2 receptor, while the cough response is mediated by other afferents expressing EP3 receptor. Our assumptions require to be verified in further study.

Previous studies have shown that postaglandins, such as PGE_2_, could activate intracellular PKA pathway in nociceptive afferents resulting in behavioral hypersensitivity [66, 67]. CAP-evoked responses in sensory neurons present robust potentiation by cAMP/PKA-dependent pathway. Treatment with PGE_2_ or activation of PKA sensitizes CAP-induced currents in rat and mouse sensory neurons [68–70]. Moreover, PKA signaling pathway has been reported to participle in EP2 receptor-mediated facilitation of vagal pulmonary C-neural response to CAP in rats [29, 30]. Owing to that PGE_2_-pretreatment enhances CAP-induced currents fully via acting EP3 receptor in vagal pulmonary C-neurons of guinea pigs (Fig 6D), we also tested whether this signaling pathway is responsible for the EP3 receptor-mediated facilitation of CAP-induced currents. We found that H-89 did not significantly affect CAP alone-induced currents (Fig 7A), similar to a previous report in which H89 failed to affect CAP-induced currents in HEK293t cells expressing wild-type TRPV1 [71]. In sharp contrast, H-89 almost blocked the PGE_2_-induced currents and its augmenting effect on CAP-induced currents. This finding, along with the full dependence of the facilitated CAP-induced currents by PGE_2_ on EP3 receptor, suggests that the PKA signaling pathway is involved in the PGE_2_ facilitation (via acting on EP3 receptor) of CAP-induced currents in vagal pulmonary C-neurons of guinea pigs.

Our results also showed that PGE_2_-induced currents were inhibited by ~33% after CAP pretreatment. A previous report has shown that incubation of both DRG neurons and TRPV1-expressing HEK293 cells with 0.1 μM CAP for 20 min induces a significant TRPV1 desensitization [72]. In agreement, we found a remarkable desensitization of vagal pulmonary C-neurons in response to the second ejection of CAP 30 s after the first one (Fig 7B). Because of the dependency of PGE_2_-induced currents on activation of TRPV1, the desensitized TRPV1 30 s after CAP pretreatment is accountable for the reduction of the subsequent PGE_2_-induced currents. In the present study, CAP pre-ejection alone blunted PGE_2_-induced currents by 33%, while that coupled with blockade of TRPV1 attenuated these currents by 69%. We surmise that this 69% attenuation results from the combination of TRPV1 desensitization (33%) induced by pre-ejection of CAP and TRPV1 blockade by pretreatment with JNJ 17203212. No attempt was made in this study to determine whether CAP pretreatment would suppress PGE_2_-induced cough because CAP/CA at the threshold concentration evokes significant bronchoconstriction and mucosal secretion [73, 74] that could confound the subsequent cough response to inhalation of PGE_2_.

Significance of our results is evident. Substantial elevation of PGE_2_ levels in the airways has been observed in a variety of diseases, including chronic obstructive pulmonary disease, cough variant asthma, idiopathic cough and cough associated with post-nasal drip, gastrooesophageal reflux disease, and eosinophilic bronchitis [75–79]. For example, sputum concentration of PGE_2_ is tripled in patients with chronic cough as compared to healthy subjects (from 0.7 to 2.1 ng/ml) [75]. Interestingly, these patients often have hypoxemia and pulmonary inflammation that could lead to certain lipoxygenase products and lactic acid, etc. in the airways to stimulate TRPV1 of PCFs [20, 80–83]. In fact, the co-presence of elevated pulmonary PGE_2_ and TRPV1 stimulants has been believed to responsible for coughs in patients with asthma, idiopathic pulmonary fibrosis, idiopathic chronic cough and COPD ([27] and for review, see [28]). Our results show that inhalation of aerosolized TRPV1 or EP3 receptor antagonist sufficiently suppresses PGE_2_-evoked cough and that 24% vagal pulmonary C-neurons co-express TRPV1 and EP3 receptor with the cross-effect between TRPV1 and EP3 receptors in the neural excitability similarly to the cough. These results suggest that a subgroup of vagal pulmonary C-neurons co-expressing TRPV1 and EP3 receptor is, at least in part, responsible for the cough response to PGE_2_. Therefore, inhalation of aerosolized TRPV1 and EP3 receptor antagonists capable of targeting vagal pulmonary sensory fibers may be an effective antitussive therapy in these patients. Further studies are required to clarify if different PGE_2_ doses or loops of vagal afferents (their second-order neurons in the NTS and/or synaptic neurotransmissions) are responsible for triggering the distinct cough patterns in response to CA/CAP and PGE_2_. Additionally, it also needs to be determined which other ion channels, in addition to TRPV1, are involved in generating PGE_2_-induced currents.

## Conclusion

**Fig 8:**
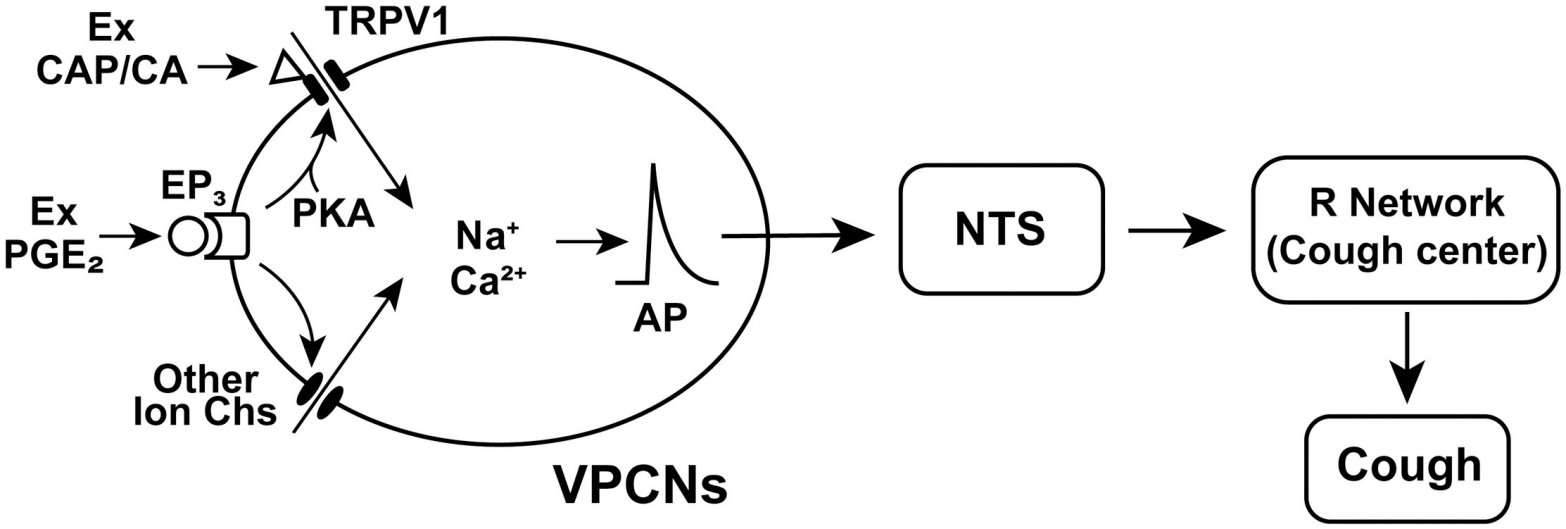
A diagram summarizing the possible pathway of cough responses mediated by EP3 receptor (TRPV1). Exogenous (Ex) rather than endogenous PGE_2_ activates EP3 receptor of vagal pulmonary C-neurons (VPCNs) to sensitize/stimulate TRPV1 via the PKA signaling pathway and other ion channels (Chs), while CAP/CA directly activates TRPV1. Once TRPV1 is activated, sodium and calcium ions flowing through TRPV1 into the neuron to cause depolarization, leading to action potential (AP) firing upon the activation of voltage-gated sodium channels. In addition, PGE_2_ may also act on other ion channels to contribute to the neural excitation. The evoked action potentials transmit in vagal afferents to promote the release of excitatory amino acids from the central terminals that stimulate the second-order neurons mainly within the nucleus tractus solitarius (NTS). These neurons further project to the respiratory network (R network) to reflexively elicit cough.

Our results in the present study are summarized in Fig 8. Exogenous rather than endogenous PGE_2_ activates EP3 receptor of PCFs (vagal pulmonary C-neurons) and thereby sensitizes/stimulates TRPV1 via PKA signaling pathway and other ion channels, while CAP/CA directly activates TRPV1.

When TRPV1 is activated, sodium and calcium ions flowing through TRPV1 into the neuron to cause depolarization, leading to action potential firing upon the activation of voltage-gated sodium channels [18, 84]. In addition, PGE_2_ may also act on other ion channels to contribute to the neural excitation. The evoked action potentials transmit through vagal afferents to promote release excitatory amino acids from their central terminals that stimulate the second-order neurons mainly within the NTS. These neurons further project to the respiratory center including cough center to reflexively elicit cough. In conclusion, the similarity of the cross-effect between TRPV1 and EP3 receptor on cough and vagal pulmonary C-neural activity observed in this study suggests that a subgroup of vagal pulmonary C-neurons co-expressing TRPV1 and EP3 receptor is, at least in part, responsible for the cough response to PGE_2_.

## Abbreviations

CA: citric acid
CAP: capsaicin
DiI, 1: 1’-dioctadecyl-3,3,3’,3’-tetramethylindocarbocyanine perchlorate
GPCR: G-protein-coupled receptor
IP: intraperitoneal
NTS: nucleus tractus solitarius
PCFs: bronchopulmonary C-fibers
PGE_2_: prostaglandin E_2_
PKA: protein kinase A
TRPV1: transient receptor potential cation channel subfamily V member 1
